# Statistical Assessment of Fracture Toughness Results from the HAZ of X80 Pipeline FCAW Girth Weld

**DOI:** 10.3390/ma15176157

**Published:** 2022-09-05

**Authors:** Hongyuan Chen, Qingshan Feng, Ying Bi, Xiongxiong Gao, Lianshuang Dai, Qiang Chi

**Affiliations:** 1 School of Materials Science and Engineering, Xi’an Jiaotong University, Xi’an 710049, China; 2 State Key Laboratory for Performance and Structure Safety of Petroleum Tubular Goods and Equipment Materials, CNPC Tubular Goods Research Institute, Xi’an 710077, China; 3 China Oil & Gas Piping Network Corporation, Beijing 100013, China; 4 Xi’an Shiyou University, Xi’an 710069, China

**Keywords:** statistical assessment, X80, pipeline, girth weld, fracture toughness

## Abstract

Due to the wide application of flux-cored arc welds (FCAW) susceptible to significant scatter in weld and Heat Affected Zone (HAZ) fracture toughness, there is an interest in methods for evaluating the reliability of welds containing defects. The mechanical properties of the FCAW girth weld of an X80 pipeline are tested and then analyzed. By obtaining fracture toughness results from a statistically significant number of SENB specimens, with notches positioned in different HAZ locations, the effect of variation within the results can be evaluated. The results of the fracture toughness tests were analyzed using statistical methods, to compare both the difference in behavior between HAZ microstructures and the variation when a similar microstructure has been sampled. The range of different characteristic toughness values was analyzed using a postulated ECA case to illustrate the sensitivity of the results to how toughness is defined. The analyses supported recommendations to be made on the optimum approach to characterizing HAZ fracture toughness for reliable pipeline assessments in multi-pass girth welds with complex HAZ microstructure distributions.

## 1. Introduction

With the wide application of high-grade pipeline steel, the risk of girth weld of pipeline failure is gradually shown. Girth weld is the weakest link of oil and gas pipelines due to its nonlinear geometry and gradient performance.

The fracture toughness of a girth weld HAZ is a combination of the effect of the parent steel composition, thickness and microstructure, and the heat input of the welding process. The latter itself is affected by the welding process, the weld joint design, and the current and voltage of the arc. The majority of pipeline steels are specified in accordance with API 5L [1]. Pipeline steels achieve their strength through microalloying along with thermomechanical controlled rolling and generally have low carbon levels. This means that the parent steels often have fine grain size and high fracture toughness. There was no significant difference in CTOD for the weld metal, whereas for the HAZ higher CTOD values were found for the 6 o’clock position [2]. Thus, further post-test fractography and metallography are required to determine the precise microstructures [3].

The challenge when welding is to keep the heat input controlled to avoid both excessive heating, which could cause grain growth along the fusion line, and avoid too low a heat input which might risk metastable hard brittle phases forming (and can cause lack of fusion flaws). Therefore, both the occurrence of high and low heat input could reduce the fracture toughness of the HAZ.

In multi-pass welds, the subsequent welding passes reheating regions of the HAZ can result in an array of different microstructures as each of the four HAZ regions is reheated within each of the regions of the subsequent HAZ. The region most susceptible to low toughness is sometimes considered to be the inter-critically reheated grain coarsened HAZ, “ICGCHAZ” [4]. However, the amount of this microstructure present also depends on the subsequent weld bead placement and the weld heat input in the joint.

When fracture toughness test results from welds and HAZs demonstrate significant scatter in their results, BS 7910 [5] suggests that post-test metallography can assist in reducing scatter in data by excluding results from the set that do not test the target microstructure. The method to carry out post-test metallography given in BS EN ISO 15653 [6], states that a notch tip within 0.5 mm ahead or to the side of the target microstructure is sufficiently close to be considered ‘valid’. However, even 0.5 mm of margin still offers scope for a range of weld and HAZ microstructures to be sampled in reality within a dataset of valid fusion line results.

All ferritic steels show fracture toughness behavior that changes with temperature; at sufficiently high temperatures, the behavior is fully ductile, while at a lower temperatures the fracture behavior becomes brittle [7]. The transition between these two behaviors occurs at the ductile-to-brittle transition temperature, and the change in fracture toughness can be a very steep gradient (although not necessarily so), while the ductile upper shelf, and the brittle lower shelf are themselves fairly shallow with temperature. This S-shape ductile-to-brittle transition curve (DBTC) is characteristic when defining the fracture behavior of steels [8].

Windle [9] proposed modeling Charpy data with the Burr distribution while Cao [10] found this approach and tanh methods show similar fitting ability for large and well-distributed datasets. The Burr distribution can fit sparse data, but the data need to be reasonably well distributed across the test temperature range encompassing the transition region. An example from the literature of typical scatter in toughness around the transition temperature is that measured from a set of over a dozen SENB tests performed at the T40 J temperatures (a characteristic transition parameter determined from a Charpy DBTC) for the HAZ of GMAW and SMAW welds in X70 steel. The results ranged from a CTOD of over 0.65 mm (fully ductile) down to less than 0.04 mm for the brittle results [11].

When steels are on the lower shelf of the DBTC, the material acts as if it has bulk brittleness and likewise, on the upper shelf, the material shows bulk general ductility. However, within the ductile-to-brittle transition, a statistical approach to brittle fracture events is necessary to describe the behavior [12]. The influence of the micro-structural dispersion on the ductile-to-brittle transition of fracture is modeled via a statistical approach by a master curve which aligns renormalized experimental data of absorbed energy or ductile fraction as a function of the temperature [13]. The distribution of microscale fracture initiation sites (such as inclusion, carbide, or grain boundary triple point) will influence whether a crack will result in cleavage fracture or not. Statistical fracture models, such as the Master Curve, have been developed to assess steels within the lower transition region and are included in standards such as ASTM E1921 [14] or BS 7910 Annex L [5]. Where mixed ductile and brittle results are obtained within a set of three fracture toughness test results, BS 7910 [5] recommends carrying out a larger number of tests. For a set of three results, all three values should be within 70% and 140% of the average value of Kmat (the equivalent for J would be for all results to be within 50% and 200% of the average J). When this scatter criterion is not met, additional test data and more advanced statistical treatment of the data are necessary to establish confidence in the choice of fracture toughness for an ECA.

Reliability describes the state of a structure performing consistently and safely. Both the safety of the structural assessment and the variation of the operating conditions and materials properties play a role. Methods to characterize the structural integrity of pipelines have existed for many years. For example, an engineering critical assessment (ECA) was carried out according to procedures from BS 7910 [5]. These assessment methods rely on the input data being accurately and conservatively characterized, and conservative.

There are scenarios where the fracture toughness from the conventional approach of using the lowest value from three equivalent standard tests might not sufficiently capture the full range of behavior to allow safe assessments to be ensured. A significant variation in fracture toughness can occur as a consequence of one or more of the following scenarios:

- The steel is within the ductile-to-brittle transition, where properties show a range of toughness values within a narrow temperature range;
- The weld comprises regions with different material properties, which are sampled within the same dataset (i.e., local brittle zones along the fusion line);
- The girth weld experiences different properties around the circumference of the weld due to the differences in welding position and heat input.

The values of fracture toughness determined during testing can be very variable, intrinsically so when steel is within the ductile-to-brittle transition, for instance. Factors such as temperature, loading rate, material thickness, microstructure, and texture can all affect the results of fracture toughness tests in nominally homogeneous materials.

Greater challenges arise when testing inhomogeneous microstructures, such as those along the fusion line in multi-pass welds. Here, each heat-affected zone (HAZ) comprises four different general bands of microstructure depending on the extent to which they have been heated. Each of these regions will be subsequently re-heated to a range of different temperatures by the subsequent weld passes. The result is a pattern of localized but very different microstructures all along the fusion line. This is also the very location where most common weld flaws will form, so it is vital to characterize the typical fracture properties that the tips of those cracks and planar flaws could experience. This variation in microstructure in the HAZ is compounded by the fact that the weld geometry and heat input varies around the circumference of girth welds fabricated in the ‘5G’ position, i.e., at the 12 o’clock position the weld position is ‘flat’ and more heat input could be experienced than in the weld at the 6 o’clock position, which is welded overhead where the heat input could be lower.

These factors contribute to the reasons for variation in fracture toughness properties in the HAZ of pipe girth welds, and this work aims to describe the magnitude of possible performance changes and to discuss their impact on service behavior. It is becoming increasingly common to take a statistical approach to determine fracture toughness for input into ECAs [15], and this work builds on those principles for fracture toughness characterization, which is based on the microstructural variation within the HAZ of an actual pipe girth weld.

## 2. Experimental Approach

### 2.1. Girth Weld Material

A pipe girth weld in X80 steel pipe with the girth welds was made using a mechanized FCAW welding process. The pipe was 12.8 mm thick with an OD of 40 in (1016 mm). The full ring had been cut into four panels for ease of transportation; the panels were centered on locations corresponding to the 12 o’clock, 3 o’clock, 6 o’clock, and 9 o’clock locations of the original weld.

### 2.2. Mechanical Test Program

**Figure 1:**
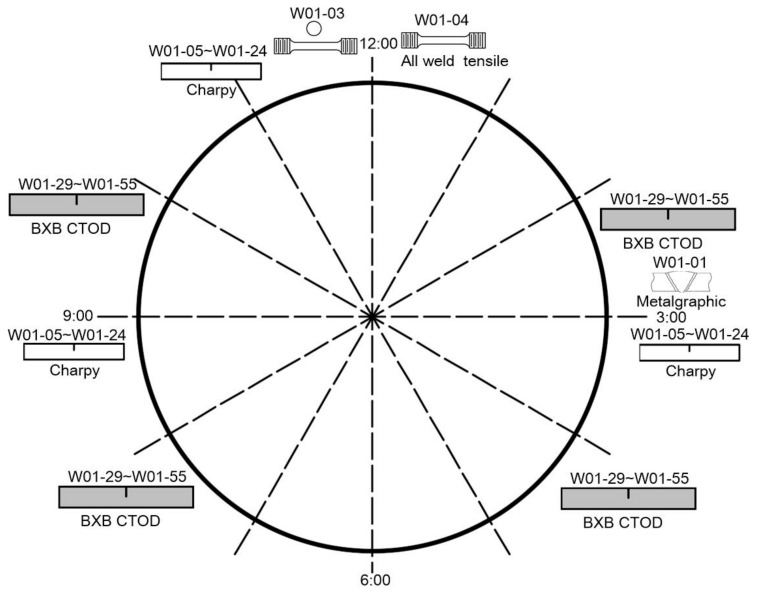
Schematic diagrams showing specimen location in the girth weld.

The girth weld panels were machined into sets of specimens for characterization, including parent and weld metal tensile tests, while some of the weld sections were used for Vickers micro-hardness mapping and for microstructural investigation. The chemical composition of the weld metal and parent metal was analyzed using optical emission spectroscopy. In addition, Charpy transition curves were generated from sets of ten specimens notched along the fusion line and along the weld centerline for specimens from the 3 o’clock, 6 o’clock, and 12 o’clock plates. From the characteristics of the weld sections, the fusion line microstructures were identified within the central part of the plate thickness, so that they could be sampled by single edge notched bend (SENB) fracture toughness test specimens notched within the standard range of a_0_/W permitted within BS EN ISO 15653 [6]. The variation within the weld bead shape along the weld seam, along with natural variation in the precise notch location, was considered sufficient to ensure the sampling range of the microstructure of the fusion line, and the ratio of crack depth to specimen width was 0/w and 0.5. A program of BxB SENB tests was carried out to allow for the range of fusion line microstructures to be sampled with sufficient repetition. The specimens were machined as close to full thickness as possible from the weld joint, allowing for full machining of the specimens. The level of misalignment in the girth weld joint often meant that, after machining, the specimens ended up being 10.5 mm to 11.5 mm in thickness. In total, 27 SENB specimens (identified as W01-29 to W01-55) were tested by sampling the fusion line. A further three SENB specimens were used to characterize the weld metal (W01-56 to -58), machined from the 9 o’clock plate. Figure 1 shows the layout of test specimens and provides details of their positions.

All fusion line specimens were subjected to post-test metallography to measure whether the distance from the fatigue pre-crack tip to the fusion line was sufficiently small to be considered a valid fusion line result, with the crack tip being no more than 0.5 mm from the fusion line, based on the measurement of the distance from the crack tip to the fusion line both ahead of the crack tip, s1, and to the side of the crack tip, s2, as described in BS EN ISO 15653 [6].

The test temperature for the SENB specimens of −10 °C was agreed based on the results of the Charpy characterization, and the anticipated service of similar pipelines. This temperature was within the ductile-to-brittle transition of the Charpy ductile-to-brittle transition curve and was intended to generate results with a range of potentially brittle and ductile behavior.

## 3. Test Results

### 3.1. Weld Sections and Microstructures

**Figure 2:**
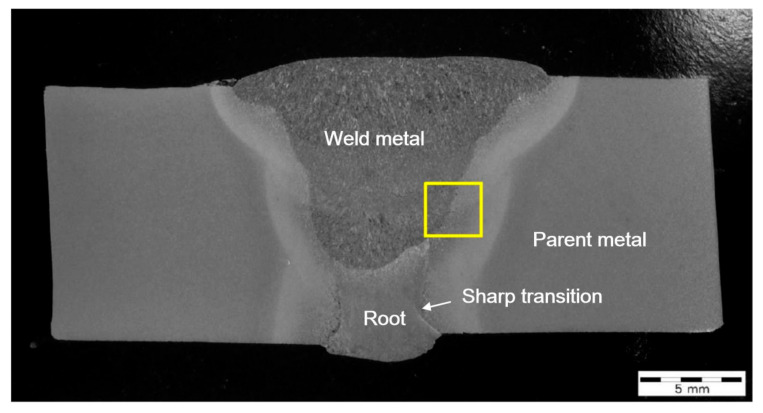
Macro section W01-04 of the 12 o’clock region of the FCAW girth weld.

The girth weld sections did not show a significant lack of root fusion flaws, despite root indications being identified from the ultrasonic testing (UT). The possible lack of side-wall fusion identified by UT appeared on the weld section to be an embedded slag inclusion within the weld metal. The root did, however, show a sharp transition on one side of the weld (Figure 2), which might be the reason for the UT root indications. There was also some axial misalignment evident from the weld sections.

**Figure 3:**
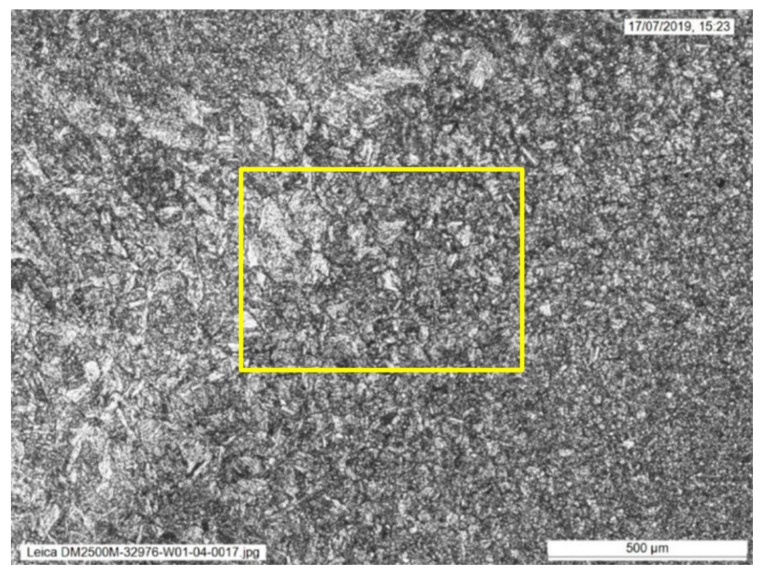
Microstructure in the location of the ICGCHAZ identified in Figure 1.

**Figure 4:**
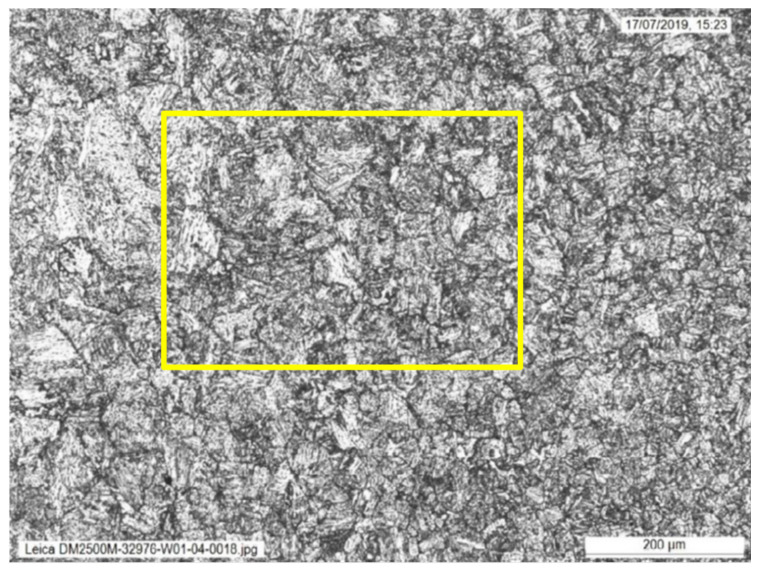
Microstructure in the location of the ICGCHAZ from Figure 2 at higher magnification, showing coarse grains decorated with second phase precipitates around the grain boundaries.

**Figure 5:**
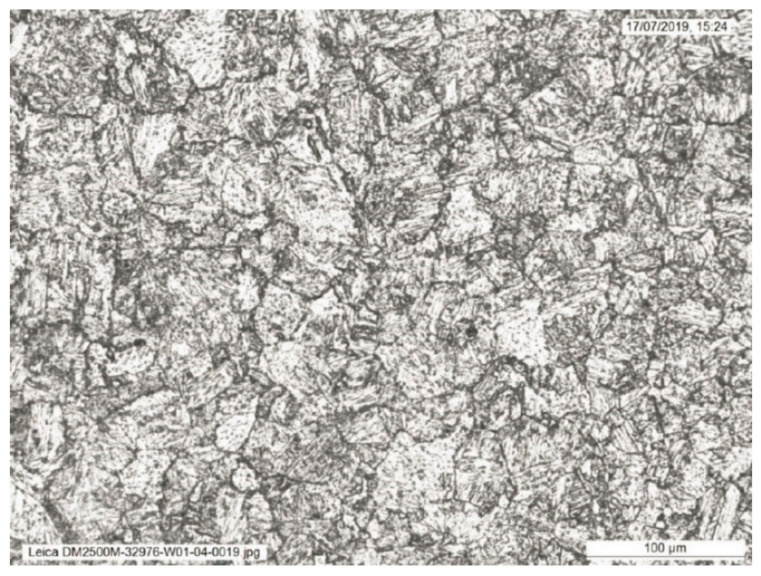
Microstructure in the location of the ICGCHAZ from Figure 3 at highest magnification, showing coarse grains decorated with second phase precipitates around the grain boundaries.

The weld sections were examined under higher magnification to investigate the microstructures along the fusion line. Specifically, areas of the grain coarsened heat affected zone (GCHAZ) that had been subsequently inter-critically reheated by the next weld pass (IC-GCHAZ) were most likely to show the lowest fracture toughness. In the IC-GCHAZ region, the coarse grains were decorated with second phase precipitates around the grain boundaries. Areas showing this kind of microstructure were identified along the fusion line around the mid-wall location of the weld (Figure 3, Figure 4 and Figure 5). However, the amount and extent of this IC-GCHAZ depends strongly on the relative bead placement and heat input. Small regions of IC-GCHAZ were seen around the mid-wall location in the sections from 12, 3, and 9 o’clock. The section from the 6 o’clock panel had a wider root and cap, and the larger fill pass provided more refinement. There was very little CGHAZ visible along the fusion line of the weld at this position. It is possible that the wider weld root and cap at this location were a response to bridging a larger fit-up gap that could have opened up while welding from 12 o’clock down to 6 o’clock on both sides of the weld.

### 3.2. Stress–Strain Curves

**Figure 6:**
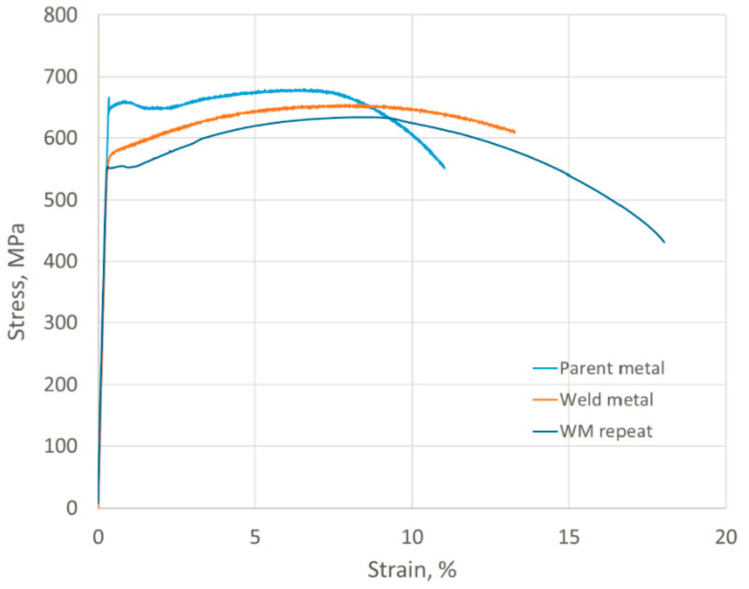
Stress–strain curves generated from round tensile specimen from the parent metal, and from two all-weld metal round tensile specimens, tested at room temperature.

The stress–strain curves from the tensile tests on the parent metal and weld metal (specimens W01-05 and W01-06 respectively) machined from the 3 o’clock plate are shown in Figure 6. The parent metal showed higher yield strength than the all-weld metal tensile, with the curves only crossing at above 8% strain when the parent metal had exceeded its UTS. A repeat of the weld metal round tensile test was carried out to confirm the result, specimen W01-76 from the 12 o’clock plate, which also showed lower strength than the parent metal.

The parent metal tensile results produced a 0.2% proof strength (Rp0.2) of 654 MPa, 0.5% extension under load yield strength (Rt0.5) of 651 MPa, and an ultimate tensile strength (UTS or Rm) of 681 MPa. The all-weld metal tensile produced an Rp0.2 of 575 MPa and a UTS of 655 MPa. The repeat all-weld tensile measured an Rp0.2 of 551 MPa and a UTS of 633 MPa, confirming the bulk undermatching of the weld metal strength in the circumferential direction compared to the parent metal in the longitudinal direction. A cross-weld tensile test with the weld cap left intact nonetheless failed away from the weld joint in the parent metal, probably as a consequence of the excess weld metal providing some reinforcement at the weld.

### 3.3. Chemical Analysis

The results of the chemical analysis are summarized in Table 1. The specifications for the API 5L X80 pipeline steel, one of the weld roots and one of the weld fill consumables, are also included for comparison. The weld metal and parent metal showed generally similar chemistry, but points of note were the higher level of manganese in the weld metal (2.06% is above the calibration range, determined by extrapolation). The weld metal composition will be affected by dilution with the parent metal, and the different compositions of the root pass and fill passes. However, the high Mn seems anomalous for the weld consumable specifications nominal compositions included in Table 1.

The parent metal had three times the amount of chromium and molybdenum than the weld metal, but it was still low levels. However, the carbon equivalent (in terms of Pcm) for the parent metal at 0.20% is higher than the weld metal at 0.18%, and might be an explanation for the under-matching weld strength. The parent steel showed very low nickel, as does the specification for the weld root consumable, yet the filler consumable specification has over 2%, resulting in a weld metal composition of less than half that.

When considering the elements that can promote good HAZ toughness, Mo, Nb and Ti are all present in the parent metal and it is reasonable to expect them to be beneficial for the HAZ.

### 3.4. Charpy Ductile to Brittle Transition Curves

The initial Charpy specimens taken from the 3 o’clock plate were used to define a ductile-to-brittle transition curve (DBTC) between −60 °C and +40 °C for specimens notched into the weld and fusion line. The subsequent DBTCs generated from sets of specimens from other pipe girth weld regions (12 o’clock and 6 o’clock) were tested at the same test temperatures, which were used to enable direct comparison.

Two of the weld metal specimens from 6 o’clock showed defects on the fracture surfaces, and so those results have been excluded from the transition curves. The small size and random orientation of these weld metal flaws (slag inclusions or small pores) meant that they were not identified from the initial manual UT on the weld.

**Figure 7:**
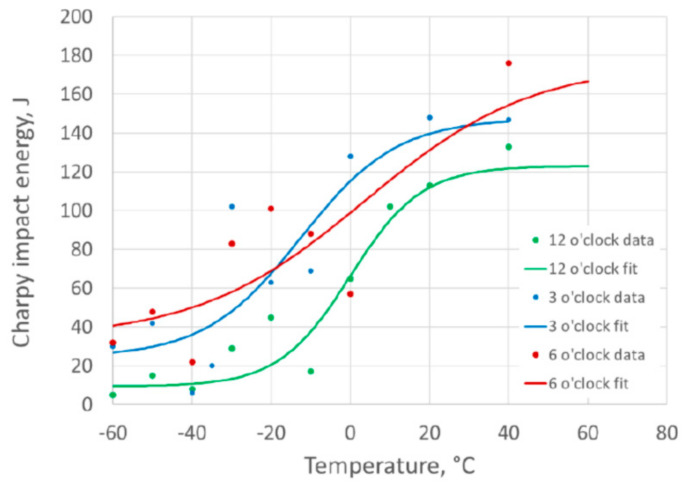
Charpy impact energy ductile-to-brittle transition curves for weld centerline notched specimens.

**Figure 8:**
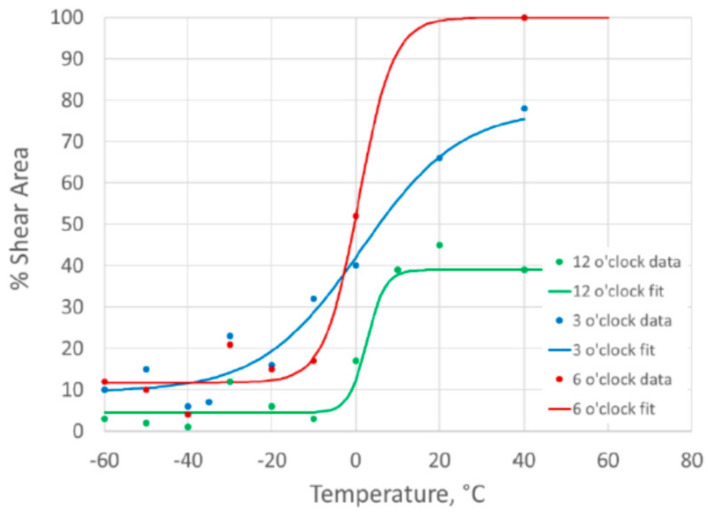
Charpy percent shear area ductile-to-brittle transition curves for weld centerline notched specimens.

**Figure 9:**
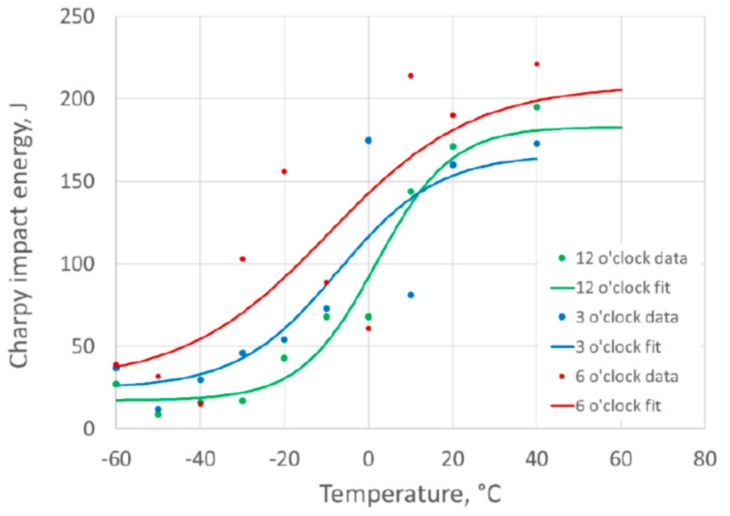
Charpy impact energy ductile-to-brittle transition curves for HAZ notched specimens.

**Figure 10:**
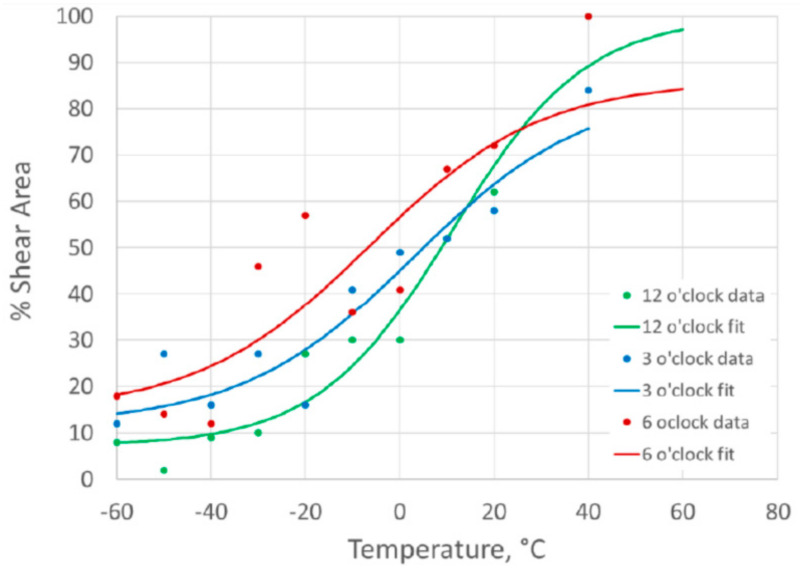
Charpy impact energy ductile-to-brittle transition curves for HAZ notched specimens.

The results and DBTCs for the weld centerline specimens are plotted in Figure 7 based on the Charpy impact energy results, and in Figure 8 based on the percentage shear area results. The results for the different clock positions around the girth weld are plotted for comparison. For the fusion line notched specimens, the DBTCs based on Charpy impact energy are given in Figure 9 and based on the percentage shear area in Figure 10.

The value of T40 J, the temperature at which a 40 J absorbed energy is predicted from the tanh DBTC fit, was determined for each of the transition curves, and the values are given in Table 2. This parameter, which is a characteristic value defining the lower part of the ductile-to-brittle transition, allows a quantitative comparison of the different DBTCs, as a higher T40 J temperature is associated with lower toughness.

There were similar transition temperatures between weld metal and fusion line notched specimens at equivalent clock positions around the girth weld, with the T40 J temperatures within about 5 °C, as shown in Table 2.

The variation around the girth weld was more significant than the difference between the weld and HAZ at any location, with the 12 o’clock location giving the highest transition temperature (lowest toughness), while the 6 o’clock location was consistently the lowest transition temperature (highest toughness). The use of the 3 o’clock location to extract the SENB specimens for characterizing the HAZ toughness was a reasonable representation of the pipe properties for this girth weld.

The upper shelf Charpy impact energy values for the weld metal (at 140–180 J) were slightly lower than for the HAZ (170 J and 220 J), and the weld metal also showed more scatter in the Charpy results than the HAZ. However, the remainder of this work is intended to concentrate on the HAZ properties, and the lower toughness of the weld metal is unusual for pipeline girth welds.

### 3.5. Fracture Toughness in Different HAZ Regions

**Figure 11:**
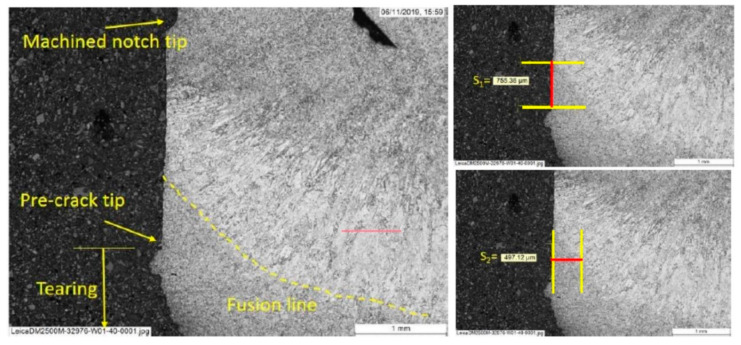
Example of post-test metallography from specimen W01-40, characterizing the distance of the crack tip from the fusion line, in terms of s1 and s2.

Of the 27 fusion line SENB specimens tested at −10°C under quasi-static conditions to BS EN ISO 15653:2018, 19 successfully sampled the target microstructure (i.e., fusion line) within 0.5 mm of the crack tip. Examples of how the values of s1 and s2 were determined from the post-test metallography are given in Figure 10 and Figure 11. Within the ‘valid’ results, two tests showed brittle behavior while 17 were maximum load fully ductile results.

**Figure 12:**
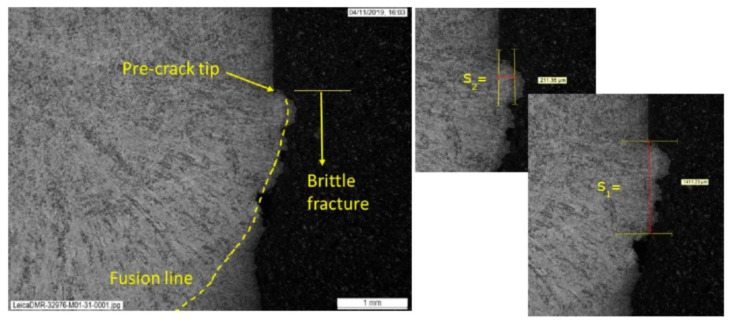
Example of post-test metallography from specimen W01-31, characterizing the distance of the crack tip from the fusion line, in terms of s1 and s2.

The weld metal fracture toughness test results are summarized in Table 3. The weld metal results were fractures with small amounts of tearing, i.e., typical of steel within the ductile-to-brittle transition regime, as predicted from the Charpy DBTCs. Values of J between only 74 and 96 kJ/m^2^ were achieved at −10°C in the weld. The weld metal on some of the post-test metallography specimens showed small pores or slag inclusions (examples can be seen in Figure 11 and Figure 12), which are likely to be the same as the flaws which were seen on the fracture surfaces of two of the weld metal Charpy specimens.

The low weld metal fracture toughness can affect the assessment of the HAZ fracture behavior, since when targeting a notch location at the fusion line, statistically, about half of the time the notch tip will sample on the weld metal side, and half the time the notch tip will sample the HAZ side of the fusion line. This was borne out from the post-test metallography, as for the specimens notched to sample the fusion line, roughly half ended up with notch tips in the weld metal and half in HAZ. Of the valid results (i.e., notch tip within 0.5 mm of the fusion line), more than half of the sampled weld metal were on the same side of the fusion line, including the two fracture results. However, the highest fracture toughness measured from a valid result was also sampling into the weld metal. Given that one of the important aspects of this work is that it represents the conditions in ‘real’ girth welds, this can be considered as another contribution to HAZ scatter.

**Figure 13:**
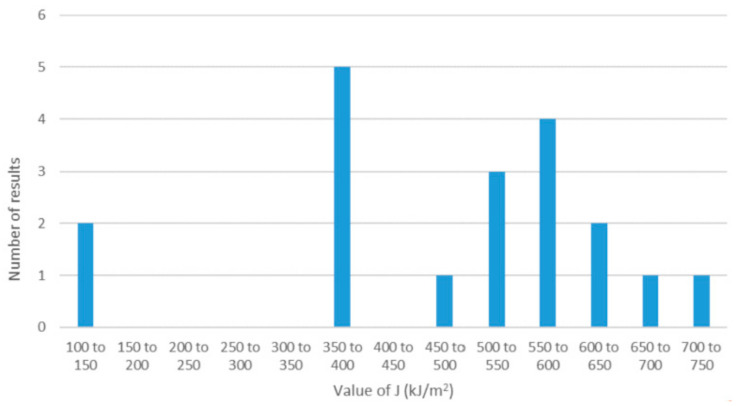
Frequency distribution of the valid fracture toughness results from the quasi-static SENB specimens notched to sample the weld fusion line, showing the range and scatter.

**Figure 14:**
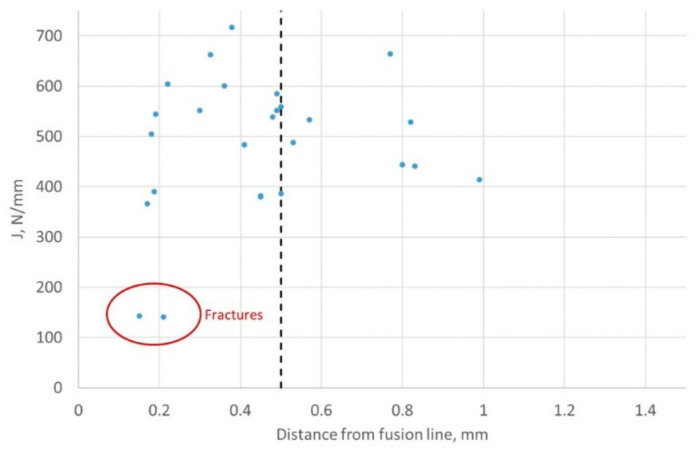
Results of quasi-static SENB specimens HAZ notched at the fusion line, plotted to show the fracture toughness, J against the shortest distance of the crack tip to the fusion line. The results to the left of the dotted line are ‘valid’.

The values of valid fracture toughness for the HAZ ranged from J of 140.6 kJ/m^2^ to 716.8 kJ/m^2^, with an average value of 478.5 kJ/m^2^. The distribution of the valid results from the fusion line notched specimens is illustrated in the bar chart in Figure 13. The HAZ results are summarized in Table 4, and the fracture toughness values, plotted against the minimum distance of the notch tip from the fusion line are illustrated in Figure 14. Despite all the specimens being marked up for notching in the same way to target the fusion line, the notch tips achieved actually sampled between 0.15 mm and 3.62 mm from the fusion line. This shows the scatter and difficulty in sampling the fusion line for fracture toughness testing of HAZs.

## 4. Significance of Scatter in Haz Toughness

### 4.1. Characterizing Fracture Toughness

A conventional way to define a ‘conservative’ value of fracture toughness, such as input to an ECA, is to test three equivalent specimens and take the lowest value of the three. With HAZ notched specimens, it is difficult to ensure that three specimens are necessarily ‘equivalent’ for the reasons outlined in Section 3. BS 7910 includes requirements for the scatter within the three results to be within the range of half to twice the average value of J (or CTOD).

The statistical implication of the minimum of three equivalent (MOTE) approach is that it is equivalent to the 50th percentile of the distribution with 87.5% confidence, or alternatively the 20th percentile with 50% confidence [15]. For a set of data larger than three results, a MOTE can also be defined, e.g., the second lowest of six results. However, the MOTE definition will be different depending on whether you assume it represents the 50th centile with 87.5% confidence, or when defined as the 20th centile with 50% confidence. The former defines MOTE as the 7th lowest of 18 results and the 11th lowest of 27 results, an approach identified here as MOTE#1. The latter, which is included in BS 7910 as “the second lowest for 6 to 10 specimens and third lowest for 11 to 15 specimens”, but also takes the fourth lowest of 18 results and fifth lowest of 27, is identified here as MOTE#2 [15].

BS 7910 [5] permits the use of MOTE#2 to process 15 data points. When there are more than 15 results, a statistical approach is taken by defining the mean and standard deviation of the dataset and defining the material toughness as the mean minus a multiple of the standard deviation. The value of k0.90 is defined based on the number of results, given in Table 7.6 in BS 7910 up to 20, and is based on defining the lower 20th percentile for the one-sided tolerance limit for a normal distribution with 90% confidence. For 19 results, k0.90 is 1.253.

It has been suggested in [15] that, for safety-critical application,s a 5th percentile approach to the determination of characteristic fracture toughness is preferred, as the 20th percentile might not provide sufficient conservatism. The equivalent k0.90 based on the 5th percentile for 19 results is 2.228 [16]. The 5th centile k0.90 for only six specimens is 3.093.

The different statistical definitions of the characteristic fracture toughness are summarized in Table 5 based on both the full set of HAZ notched specimens and the sub-set of valid specimens with notch tips within 0.5 mm of the fusion line. When only the data from specimens with valid notch positions are considered, the MOTE from both methods is similar, giving J between 380–390 kJ/m^2^. The mean minus k0.90 of the standard deviation (M-k0.90SD) for the 20th percentile is lower at 284 kJ/m^2^, while M-k0.90SD for the 5th percentile is only 124.6 kJ/m^2^.

If HAZ notched tests are carried out, but without post-test metallography to confirm which results are valid or not (equivalent to taking the full dataset here), the equivalent values of toughness are predicted to be slightly higher than they should be if invalid results are discarded.

The fusion line SENB results were also divided into nine sets of three, to illustrate that the effect of the toughness that would have been identified had only three tests. The lowest value of the set of three is given in Table 6, along with whether the scatter within the set of three would meet the requirements in BS 7910 for the data to be within half to twice the average. Within the set of three results, it is also noted whether the minimum value would have been a valid result (notch within 0.5 mm of the fusion line). In all the sets of three, the minimum value was also a valid result, but most sets did not comprise three valid results. The MOTE from the different sets of three results varied from 140.6 kJ/m^2^ up to over 500 kJ/m^2^, even when the sets of three were selected only from valid fusion line results. However, when the valid results were assessed as sets of six, and MOTE of the second lowest of six results was applied, the three sets of six results all produced MOTE values between 380 and 390 kJ/m^2^ (Table 7); this is much more consistent with the characterization and with the MOTE for the full set of valid data.

### 4.2. ECA Cases

To illustrate the effect of scattering in HAZ toughness, and how the data are used to characterize fracture toughness, an Engineering Critical Assessment (ECA) was carried out using a set of conditions representative of the service for these kinds of FCAW girth welds in an X80 line pipe. Pipe dimensions were assumed to be 1016 mm OD and a 12.8 mm wall thickness, with the girth weld having a weld cap width of 15 mm. The applied stress was based on a nominal value of half of SMYS (278 MPa), to ensure the assessment remained stress-based rather than strain-based, which would not be permitted for under-matching welds.

BS 7910 advises that the lower strength of the parent and weld metal is used for assessing the HAZ so, in this case, the weld metal properties were used to enable a case loaded to the stress level given to allow sensitivity studies on fracture toughness. The tensile properties of the weld metal were used, based on the stress–strain curve from specimen W01-76, producing a yield strength of 551 MPa and UTS of 633 MPa.

No additional stress concentration factor due to misalignment was added for this example case. The weld was assumed to be in the as-welded condition with residual stress based on the assumed tensile properties. It is important to note that this case is intended to illustrate the sensitivity of the result to fracture toughness assumptions, rather than to predict safe conditions for a girth weld. Changing the value of fracture toughness will move the assessment point along the vertical (Kr) fracture axis of the failure assessment diagram (FAD).

**Figure 15:**
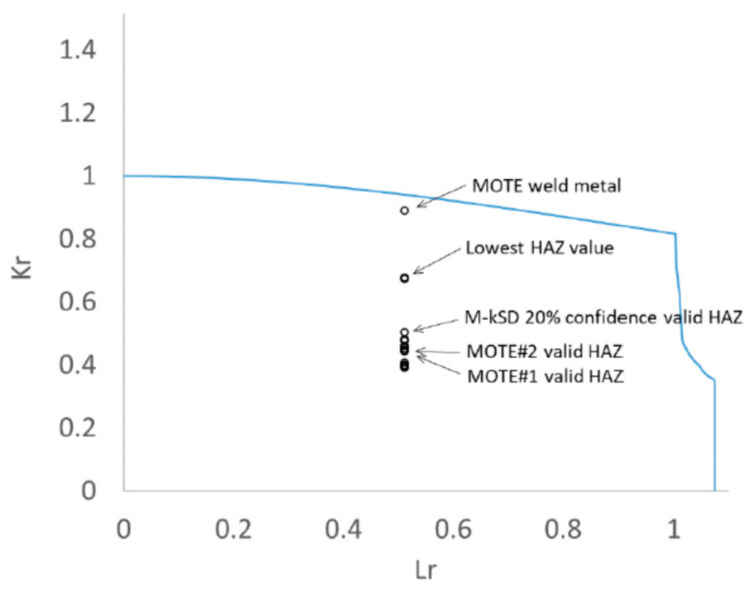
Failure Assessment Diagram (FAD) showing example pipe girth weld assessments with sensitivity cases for a range of fracture toughness values based on interpretation of these experimental results listed in Table 8.

For an assumed external surface breaking flaw of 5 mm deep and 25 mm long, sensitivity cases were based on the range of characteristic values of toughness that could have been defined for this HAZ from valid specimens sampling the fusion line, from 140.6 kJ/m^2^ to 539.1 kJ/m^2^. The results are illustrated in Figure 15 and summarized in Table 8.

The equivalent assessment is based on the weld metal data; both the full set and just the valid data are included for comparison. The lowest fracture toughness obtained by the weld metal and by the fusion line make these cases the worst-case scenarios for the pipe girth weld structural integrity.

However, considering the assessment based on the fusion line fracture toughness, the lowest toughness values of 124.6 kJ/m^2^ from the 5th centile M-k0.9SD, and 140.6 kJ/m^2^ and 142.6 kJ/m^2^ from sets of three with excessive scatter, can be considered over-conservative as the scatter is more than what is permitted in BS 7910, and these produced the highest values of Kr. The majority of the sensitivity cases produced Kr values between 0.44 and 0.50, including the 20th centile M-k0.90SD and the MOTE when applied to valid data (both of these methods are given in BS 7910 and gave Kr values of 0.504 and 0.447 respectively).

## 5. Conclusions

The results of the fracture toughness tests carried out on this girth weld were analyzed by statistical methods, to compare both the difference in behavior between HAZ and weld microstructures, but also the variation when a similar microstructure has been sampled. These differences were illustrated quantitatively by a postulated ECA case, in order to make recommendations on the optimum approach to characterizing HAZ fracture toughness for reliable pipeline assessments in multi-pass girth welds with complex HAZ microstructure distributions.

To capture the behavior of the HAZ—even though it is intrinsically variable—it is necessary to ensure the specimen notches are sampling sufficiently close to the fusion line. Although post-test metallography can add to the cost of performing fracture toughness testing, where complex microstructural regions within the HAZ can dominate the behavior of the whole joint, understanding what has actually been sampled is important. In this work, the outer HAZ generally had higher toughness, while the weld metal had lower toughness. Some of the specimen notches within 0.5 mm of the fusion line sample the outer edge of the weld metal, which might account for some of the scatter of the lowest toughness results. The range of HAZ fracture toughness results obtained in this work does not show a bell-shaped ‘normal’ distribution (Figure 13), which suggests sources of scattering beyond typical material variation. The influencing factor could be the weld metal sampled at a location with lower toughness, and the local areas of coarse grain heat affected zone ICGCHAZ are sampled. The existence of these areas may also change due to the surrounding weld location and the test temperature within the ductile–brittle transition range. However, the definitions based on M-k0.90SD assume the properties are described by a normal distribution, and where the results are not normally distributed, gathering a larger dataset will provide better characterization.

In order to ensure a sufficient number of valid results, for HAZs, it is recommended to test a larger number of specimens. Ideally, at least six valid results would be used and the second-lowest of the six results would characterize the ‘minimum of three equivalent’ value. This means that a larger number of tests would be necessary to account for those where the notch is not close enough to the target microstructure.

For this particular flux-cored arc weld, it is the weld metal properties that have the strongest influence on the structural integrity. The fracture toughness of the weld is low at a temperature of −10 °C; lower than the HAZ results. It also shows weld metal strength under-matching compared to the parent metal from round tensile tests and micro-hardness mapping, although the failure location from the cross-weld tensile test was remote from the weld joint. Despite the purpose of this work being to look at HAZ properties, it is important to recognize the concerns about the weld metal that the results have identified in terms of under-matching strength and fracture toughness compared to the HAZ. It is recommended that a further review or investigation of the FCAW welding procedure, and weld metal properties, is carried out. The high yield-to-tensile ratio of the parent pipe is another issue identified by this work that affects the overall integrity of these girth welds. If an FCAW girth weld for strain-based service loading conditions was expected to develop, it is vital that the weld metal has overmatching strength and high fracture toughness in terms of tearing resistance to enable a robust fitness-for-service assessment to be made.

Based on the results and analysis carried out on the HAZ of this FCAW girth weld, the following overall conclusions are:

1. Even when a set of HAZ specimens are all notched by the same method, it is unlikely that they will all sample sufficiently close to the target microstructure. Post-test metallography is necessary to identify which are valid;
2. Including results that had not been sampled within 0.5 mm of the fusion line meant that the characteristic HAZ toughness was over-estimated when using ‘minimum of three equivalent (MOTE)’ and statistical methods, compared to the situation when only valid data were used;
3. The MOTE when applied to a set of six valid results provides a much more consistent characteristic toughness than when sets of only three specimens are used. The MOTE from sets of six results were similar to both MOTE approaches and when applied to the full set of 19 valid results; HAZ in this FCAW was 380–390 kJ/m^2^;
4. The fracture toughness value given by the statistical method of the 20th percentile with 90% confidence (as shown in BS 7910) is slightly lower; however, taking the failure assessment diagram as an example, the results are similar to the MOTE method.

Based on the conclusions obtained from the research, the recommendations for statistical assessment of fracture toughness results from HAZ of an X80 pipeline FCAW girth weld can be given as follows:

1. To characterize the fracture toughness of HAZs, generate at least six valid test results, which may require testing nine to twelve initially to account for scattering in the eventual notch tip position in relation to the fusion line;
2. Carry out post-test metallography when sampling the HAZ to ensure valid results can be identified from the full dataset.
3. Either the MOTE approach or the mean minus k0.90 approach from BS 7910 can be applied to determine the fracture toughness for a dataset between six and nineteen data points to give similar results in an ECA case.

## Figures and Tables

**Table 1:** Chemical composition of the parent metal and weld metal in the X80 FCAW based on analysis made using optical emission spectroscopy, along with specified compositions for parent and weld filler me.

Element	Parent Metal	Weld Metal	API 5L X80 (Max)	E7016 ** (Weld Root)	E81T8-Ni2 J H8 *** (Weld Fill)
C	0.06	0.04	0.12	0.04–0.08	0.05
Si	0.23	0.19	0.45	0.39–0.67	0.05
Mn	1.82	2.06 *	1.85	1.10–1.60	1.28
P	0.01	0.008	0.025	0.005–0.020	0.009
S	0.003	0.004	0.015	0.004–0.012	0.004
Cr	0.3	0.097	0.50	0.01–0.07	
Mo	0.2	0.053	0.50	0.01–0.03	
Ni	0.006	0.7	1.00	<0.07	2.34
Al	0.04	0.69			0.93
As	0.01	0.005			
B	<0.0005	0.0006			
Cu	0.016	0.04			
Nb	0.077	0.013	≤0.15 combined		
Ti	0.017	0.008			
V	0.004	0.005		0.01–0.02	
Sn	<0.004	0.006			
Zr	<0.005	0.06			
Ca	0.0016	<0.0003			
PCM	0.196	0.176	0.25		

* Above the calibration range and hence determined by extrapolation. ** Lincoln Pipeliner 16P. *** Hobart Fabshield X80.

**Table 2:** Charpy T_40 J_ transition temperatures for girth weld and HAZ at different clock positions.

T_40 J_, °C	12 o’Clock	3 o’Clock	6 o’Clock
Weld centerline	−9.0	−36.0	−62.5
HAZ Fusion line	−14.7	−32.3	−55.7

**Table 3:** Results of weld metal centerline fracture toughness tests.

Specimen No.	Test Temperature, °C	CTOD, mm	J, mm Result	Result
W01-56	−10	0.104	83.66	δ/Ju fracture
W01-57	−10	0.126	96.45	δ/Ju fracture
W01-58	−10	0.097	73.96	δ/Ju fracture

**Table 4:** Results of fusion line fracture toughness tests carried out at −10 °C, including the distances of the crack tip from the fusion line, both in the crack plane (s1) and parallel to the crack if measured (s2). Also indicated is whether the notch tip was located on the weld metal side of the fusion line or the HAZ side of the fusion line.

Specimen No.	CTOD, mm	J, mm	Result	S1, mm	S2, mm	Sampled Target Region?	Notch Tip Side of the Sampled Target Region?
W01-29	0.498	414.7	δ/Jm max load	1.66	0.99	N	HAZ
W01-30	0.445	380.2	δ/Jm fracture	0.66	0.45	Y	HAZ
W01-31	0.162	140.6	δ/Ju fracture	1.41	0.21	Y	Weld Metal
W01-32	0.675	664.2	δ/Jm max load	1.56	0.77	N	HAZ
W01-33	0.791	487.9	δ/Jm max load	0.73	0.53	N	Weld metal
W01-34	0.599	381.6	δ/Jm max load	1.25	0.45	Y	Weld metal
W01-35	0.553	504.7	δ/Jm max load	0.18	N/A	Y	Weld metal
W01-36	0.669	600.6	δ/Jm max load	0.36	N/A	Y	Weld metal
W01-37	0.559	559.1	δ/Jm max load	2.46	0.50	Y	HAZ
W01-38	0.580	551.7	δ/Jm max load	0.3	N/A	Y	Weld metal
W01-39	0.567	483.1	δ/Jm max load	0.61	0.41	Y	Weld metal
W01-40	0.419	386.3	δ/Jm max load	0.76	0.50	Y	HAZ
W01-41	0.418	366.3	δ/Jm max load	0.17	N/A	Y	Weld metal
W01-42	0.589	544.4	δ/Jm max load	0.19	N/A	Y	Weld metal
W01-43	0.552	604.3	δ/Jm max load	0.48	0.22	Y	HAZ
W01-44	0.167	142.6	δ/Jc fracture	0.15	N/A	Y	Weld
W01-45	0.555	529.1	δ/Jm max load	1.92	0.82	N	HAZ
W01-46	0.596	533.2	δ/Jm max load	1.85	0.57	N	HAZ
W01-47	0.576	552.0	δ/Jm max load	1.67	0.49	Y	HAZ
W01-48	0.623	584.7	δ/Jm max load	2.74	0.49	Y	Weld metal
W01-49	0.641	679.9	δ/Jm max load	3.62	N/A	N	HAZ
W01-50	0.502	440.8	δ/Jm max load	1.06	0.83	N	HAZ
W01-51	0.519	443.7	δ/Jm max load	2.15	0.80	N	HAZ
W01-52	0.496	390.3	δ/Jm max load	0.313	0.187	Y	Weld metal
W01-53	0.758	662.2	δ/Jm max load	0.406	0.326	Y	HAZ
W01-54	0.828	716.8	δ/Jm max load	0.379	N/A	Y	Weld metal
W01-55	0.468	539.1	δ/Jm max load	0.616	0.479	Y	HAZ

**Table 5:** Statistical definitions of fracture toughness, J, based on both the full set of HAZ notched specimens, and on the sub-set of valid specimens with notch tips within 0.5 mm of the fusion line.

J (kJ/m^2^)	Valid FL Notched Data (19 Results)	All HAZ Data (27 Results)
Mean	478.5	492.0
Standard deviation	155.2	140.9
Median	504.7	529.1
MOTE#1	390.3	483.1
MOTE#2	380.2	381.6
Mean minus 1 StDev	323.3	351.1
Mean minus 2 StDev	168.1	210.3
Mean minus k0.90 StDev (20th percentile) *	284.0	317.3
Mean minus k0.90 StDev (5th percentile) **	124.6	196.2

* Recommended method in BS 7910:2019 for dataset larger than 15 results. ** Suggested conservative approach for safety critical structures from Pisarski, 2017.

**Table 6:** Examples of results from separate batches of three specimens, taken from the full dataset, and from the subset of valid results.

Batch of Three Specimens	Specimen Numbers	Lowest of Three Values of J, kJ/m^2^	All Results between 0.5 and 2 × Mean	Notch ≤ 0.5 mm of FL	Number of Valid Results
First	29 to 31	140.6	N	Yes	2 of 3
Second	32 to 34	381.6	Y	Yes	1 of 3
Third	35 to 37	504.7	Y	Yes	All 3
Fourth	38 to 40	386.3	Y	Yes	All 3
Fifth	41 to 43	366.3	Y	Yes	All 3
Sixth	44 to 46	142.6	N	Yes	1 of 3
Seventh	47 to 49	522.0	Y	Yes	2 of 3
Eighth	50 to 52	390.3	Y	Yes	1 of 3
Ninth	53 to 55	539.1	Y	Yes	All 3
All valid-1	30, 31, 34	140.6	N	/	/
All valid-1	35–37	504.7	Y	/	/
All valid-1	38–40	386.3	Y	/	/
All valid-1	41–43	366.3	Y	/	/
All valid-1	44, 47, 48	142.6	N	/	/
All valid-1	52–54	390.3	Y	/	/

**Table 7:** Examples of results from separate batches of six valid specimens.

Batch of Three Specimens	Specimen Numbers	Lowest of Three Values of J, kJ/m^2^
First	30–31, 34–36	380.2
Second	37–42	386.3
Third	43–44, 47–48, 52–53	390.3

**Table 8:** Summary of ECA sensitivity cases.

J Value, kJ/m^2^	SOURCE	Kr	Inside the FAD
76.0	MOTE weld metal data	0.890	Y
124.6	Mean–K0.90 StDev 5th percentile valid data	0.715	Y
140.6	Lowest of 3 results (excessive scatter)	0.678	Y
142.6	Lowest of 3 results(excessive scatter)	0.674	Y
168.1	Mean–2 StDev valid data	0.628	Y
196.2	Mean–K0.90 StDev, 5th percentile All data	0.588	Y
210.3	Mean–2 StDev All data	0.571	Y
284.0	Mean–K0.90 StDev, 20th percentile valid data *	0.504	Y
317.3	Mean–K0.90 StDev, 20th Percentile all data	0.481	Y
323.3	Mean–1 StDev valid data	0.478	Y
351.1	Mean–1 StDev all data	0.462	Y
366.3	Lowest of 3 results example	0.454	Y
380.2	MOTE#2 valid data *	0.447	Y
381.6	MOTE#2 all data/Lowest of 3 results	0.447	Y
386.3	Lowest of 3 results example	0.445	Y
390.3	MOTE#1 valid data/Lowest of 3 results	0.443	Y
483.1	MOTE#1 all data	0.407	Y

* Approach included in BS 7910.
